# Brazilian chronic dialysis survey 2017

**DOI:** 10.1590/2175-8239-JBN-2018-0178

**Published:** 2019-03-28

**Authors:** Fernando Saldanha Thomé, Ricardo Cintra Sesso, Antonio Alberto Lopes, Jocemir Ronaldo Lugon, Carmen Tzanno Martins

**Affiliations:** 1 Universidade Federal do Rio Grande do Sul Porto AlegreRS Brasil Universidade Federal do Rio Grande do Sul, Porto Alegre, RS, Brasil.; 2 Universidade Federal de São Paulo São PauloSP Brasil Universidade Federal de São Paulo, São Paulo, SP, Brasil.; 3 Universidade Federal da Bahia SalvadorBA Brasil Universidade Federal da Bahia, Salvador, BA, Brasil.; 4 Universidade Federal Fluminense NiteróiRJ Brasil Universidade Federal Fluminense, Niterói, RJ, Brasil.; 5 Sociedade Brasileira de Nefrologia São PauloSP Brasil Sociedade Brasileira de Nefrologia, São Paulo, SP, Brasil.

**Keywords:** Kidney Failure, Chronic, Renal Dialysis, Census Data, Epidemiology

## Abstract

**Introduction:**

Having national data on chronic dialysis is essential in treatment planning.

**Objective:**

To present data of the survey from the Brazilian Society of Nephrology on patients with chronic kidney disease on dialysis in July 2017.

**Methods:**

Data was collected from dialysis units in Brazil. The data collection was done using a questionnaire completed online by the dialysis units.

**Results:**

Two hundred and ninety-one centers (38.4%) answered the questionnaire. In July 2017, the estimated total number of dialysis patients was 126,583. National estimates of prevalence and incidence rates of dialysis patients per million population (pmp) were 610 (range: 473 in the North region and 710 in the Midwest) and 194, respectively. The incidence rate of new dialysis patients with diagnosis of diabetic nephropathy was 77 pmp. The annual gross mortality rate was 19.9%. Of the prevalent patients, 93.1% were on hemodialysis and 6.9% on peritoneal dialysis, with 31,226 (24%) on the waiting list for renal transplantation. Venous catheter was used as access in 22.6% of patients on hemodialysis. The prevalence rate of positive serology for hepatitis C continued with a tendency to decrease (3.3%).

**Conclusion:**

The absolute number of patients and rates of incidence and prevalence on dialysis continued to increase; the mortality rate tended to rise. There were obvious regional and state discrepancies in these rates.

## Introduction

For the past nine years, the Brazilian Society of Nephrology (SBN) has been performing an online annual national survey, gathering information about patients on chronic dialysis from registered active renal care centers. These epidemiologic and technical data are useful for policy makers, the government, health providers, patient care, and academic purposes. Despite the difficulties inherent to surveys based on voluntary data submission, a significant portion of renal care centers in Brazil has joined the effort.

This paper describes the characteristics of chronic dialysis patients in Brazil on July 1^st^ 2017. It also presents trend data from chronic dialysis programs in Brazil in the 2013-2017 period, including the incidence of patients on chronic dialysis with baseline diseases related to *diabetes mellitus* and an estimate of dialysis prevalence per state.

## Methods

A survey was conducted on the second semester of 2017 to collect data from chronic dialysis patients in outpatient settings seen in all centers registered with the SBN. From August to December 2017, the survey was available on the SBN website and all dialysis centers were invited by letter and e-mail to answer the questionnaire and submit their data electronically to SBN.

Invitations were sent repeatedly every month to the centers that had not submitted their data until the submission deadline (Dec 31, 2017). The chairpersons of the SBN regional centers were asked to contact the directors of the dialysis centers in their regions to reinforce the invitation to fill out the survey. In December 2017, the SBN secretariat called the centers that had not responded to ask them to do it. The questions concerning most of the sociodemographic, clinical, workup, and treatment variables reflected the realities of patients on dialysis on July 1, 2017. Data concerning death rates and new patients starting dialysis were referred to July 2017 and were estimated for the year.

The SBN had 852 registered outpatient dialysis centers in July 2017, of which 758 had active chronic dialysis programs; of these, 291 (38.4%) responded the survey and had their data analyzed. The data submitted by the participating centers covered 48,596 patients on dialysis. The data sets submitted by the centers were grouped to avoid portraying individual patient information and, therefore, were interpreted as the representation of the average patient and average treatment in each dialysis center.

National data were estimated based on the numbers expected for non-participating centers and their locations. Non-participating centers were assigned the mean number of patients expected for their respective regions, and their numbers were thus included in the national estimates.

The estimates for the Brazilian population and the numbers for each region of the country updated for July 2017 used in the prevalence and incidence calculations were borrowed from the Brazilian Institute of Geography and Statistics (IBGE). According to the IBGE, the Brazilian population in July 2017 was 207.66 million inhabitants. Grouped data were used to estimate the proportion of patients failing to meet the recommended targets^1^^,^^2^ for dialysis dose (Kt/V or urea reduction ratio) and serum levels of albumin, phosphorus, PTH, and hemoglobin. Most of the data are shown in descriptive form and refer to 2017; they were compared to data from previous years.^3^^,^^4^^,^^7^

## Calculations performed in estimations

Total estimated number (N) of patients on July 1: number of patients in the sample/proportion of participating centers. Estimated global prevalence: Total estimated N of patients on July 1/Brazilian population on July 1, 2017, reported in per million population (pmp).

In the regional and state estimations of N and ratios, the data considered were restricted to specific regions or states. Estimated total N of patients initiating treatment in 2017: (informed N of individuals starting treatment in July x 12)/proportion of active participating centers. Estimated global incidence: estimated total N of patients starting treatment in 2017/Brazilian population on July 1, 2017, reported in pmp.

The prevalence rates concerning demographic, clinical, workup, and medication variables were reported in relation to the total values derived from the answers related to each of the investigated factors among the 48,596 patients seen in the participating centers. Estimated total N of deaths in 2017: (N of deaths reported in July x 12)/proportion of active participating centers. Gross death rate: total estimated N of deaths in 2017/estimated N of patients on dialysis on July 1, 2017.

## Results

**Figure 1:**
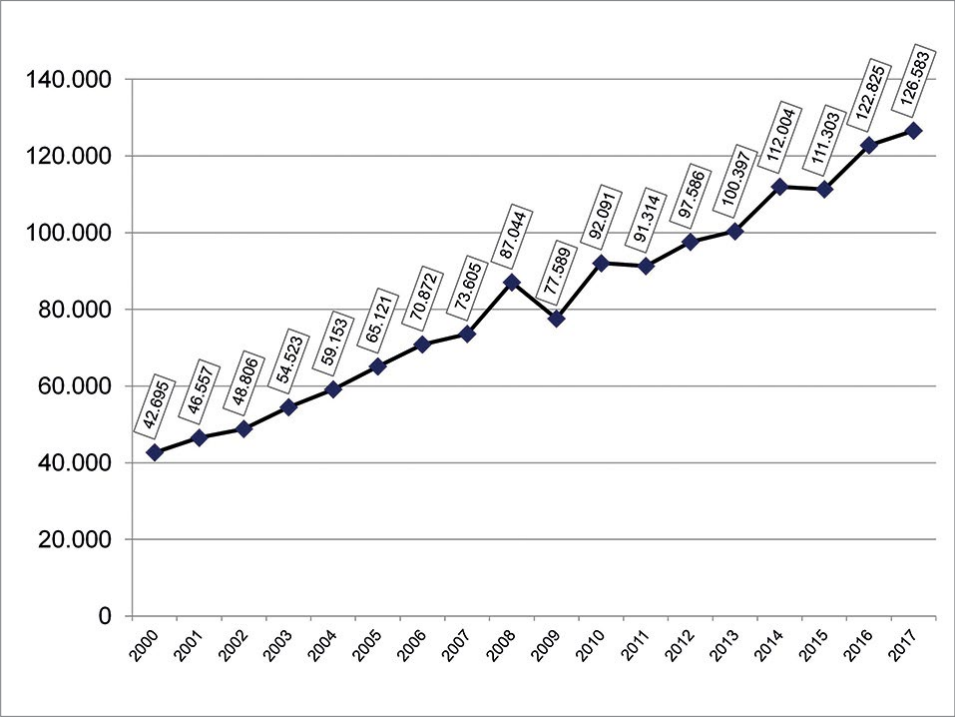
Estimated number of patients on dialysis per year.

The total number of active dialysis centers increased 37.8% from 550 in 2002 to 758 in 2017, whereas the number of patients increased 159.4% in the same period (Figure 1). The distribution of active centers was 46% in the Southeast region, 20% in the South, 10% in the Midwest, 19% in the Northeast and 5% in the North. Participating centers (n=291) were 38.4% of active centers, ranging from 24 % in the Midwest to 44% in the Southeast. Participating centers were mostly private (70%), and non-academic (85%). Fifty percent of them were located outside hospitals, and 73% were entitled to attend patients from both the public health system and private health insurance companies.

A total of 48,596 patients were receiving treatment in the 291 participating centers. Eighty-two percent of patients were supported by the Brazilian public healthcare system (SUS) and 18% by private health insurance (it was 15% in 2014). The dialysis centers had an occupation rate of 85%. Eighty-three percent of the centers had patients with chronic kidney disease under conservative management and 73% treated patients with acute kidney injury. The reported number of nephrologists working at the participating centers (n=1,731) indicated that each nephrologist took care of 28 dialysis patients on average. This proportion was similar in all regions except in the North region with 1 nephrologist for 44 patients. The question about the length of time dialysis machines were used showed that the proportion of machines with less than one year of use dropped from 13% in 2014 to 9% in 2017, whereas the proportion with more than 6 years of use increased from 37% to 44% in the same period.

**Figure 2:**
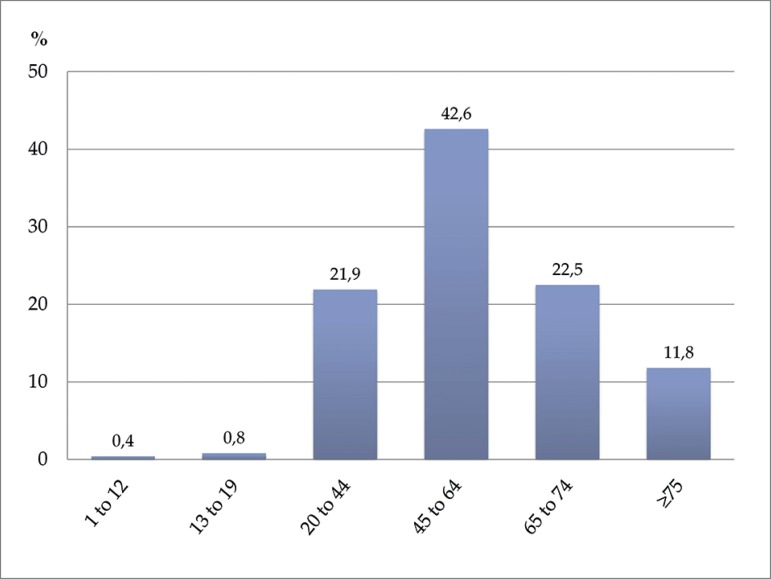
Proportion of patients on dialysis according to age group (years).

**Figure 3:**
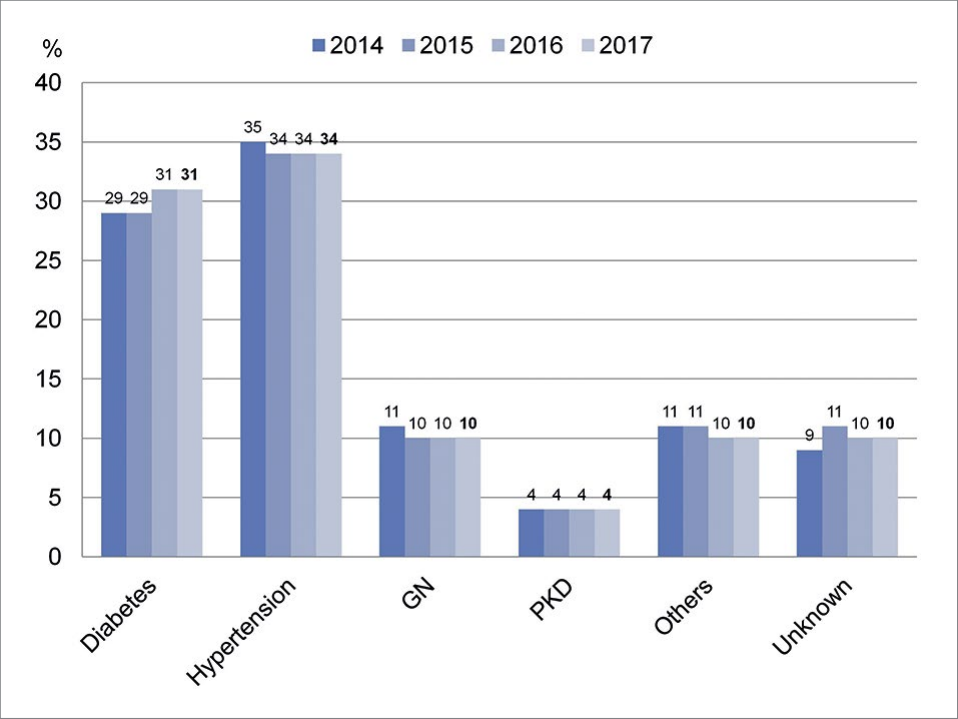
Basic diagnosis of patients on dialysis.

The total number of patients on dialysis in Brazil on July 1, 2017 was estimated at 126,583 (Figure 1). This number indicates an increase of 3,758 patients in one year (3%). If we compare three 5-year periods from 2002 to 2017, the average annual increase of patients was 4,960 from 2002 to 2007 (approximately 51% in five years), 4,796 from 2007 to 2012 (32.6%), and 5,799 from 2012 to 2017 (29.7%). Peritoneal dialysis was used by 6.9% of patients, the majority of whom were on automated peritoneal dialysis (APD); frequent (> 4 times a week) hemodialysis by 1.3%, and conventional hemodialysis by 91.8%. Again, the proportion of peritoneal dialysis patients covered by private health insurance (7.6%) was greater than those covered by SUS (6.7%). Also, frequent hemodialysis was more common in private health insurance (6.1%) than SUS (0.2%) patients. Age distribution is shown in Figure 2; 58% of patients were male. The most frequent primary causes of end-stage CKD in 2017 were hypertension (34%) and diabetes (31%). No significant change has been observed in primary diagnoses over the last few years (Figure 3).

Body mass index (BMI) was stratified in five ranges, and the proportion of patients in each stratum has not changed significantly since 2014. In 2017, 9% had BMI < 18.5 kg/m^2^; 50% were between 18.5 and 24.9 kg/m^2^, 28% between 25 and 29.9 kg/m^2^, 12% between 30 and 39.9 kg/m^2^, and 1%≥ 40 kg/m^2^.

**Figure 4:**
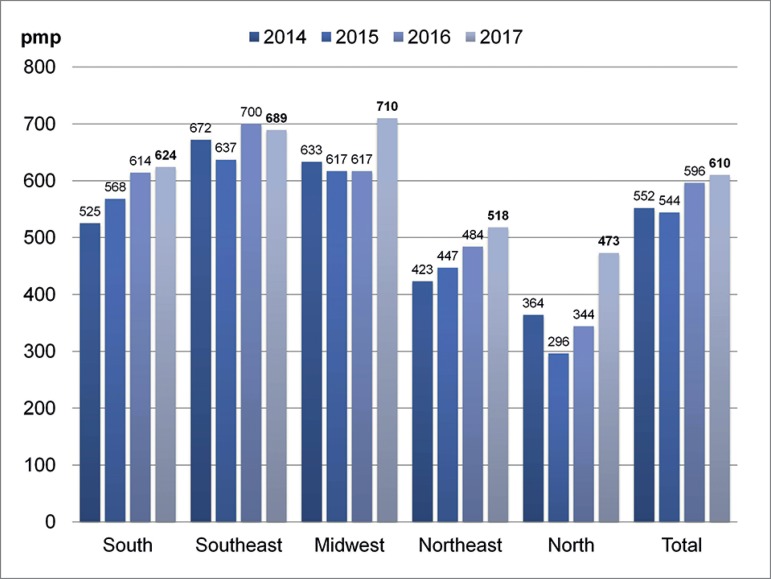
Estimated prevalence of patients on dialysis per region.

The estimated prevalence of dialysis in 2017 was 610 patients pmp, with rates ranging from 473 pmp in the North to 710 pmp in the Midwest (Figure 4). Prevalence tended to increase in all regions along the years. It increased globally from 475 pmp in 2011 to 610 pmp in 2017 (28.4%), an annual increase of 22.5 pmp. Table 1 shows the estimates for absolute numbers and prevalence per state on July 1, 2017. Most patients were on dialysis in the states of São Paulo (30,274), Minas Gerais (15,295), and Rio de Janeiro (10,578); prevalence greater than 700 patients pmp was observed in Alagoas (864), Minas Gerais (724), and Distrito Federal (712).

**Table 1:** Estimated number and prevalence of dialysis patients per State in 2017

State	Total	Prevalence/pmp	State	Total	Prevalence/pmp
AC	78	94	PB	1386	344
AL	2917	864	PE	6001	633
AM	932	229	PI	*	*
AP	*	*	PR	7522	664
BA	7953	518	RJ	10578	633
CE	5733	636	RN	1796	512
DF	2164	712	RO	*	*
ES	2072	516	RR	*	*
GO	3312	489	RS	7550	667
MA	2011	287	SC	3325	475
MG	15295	724	SE	786	344
MS	*	*	SP	30274	671
MT	2081	622	TO	*	*
PA	4714	563			

pmp: per million population

* Not enough data.

**Figure 5:**
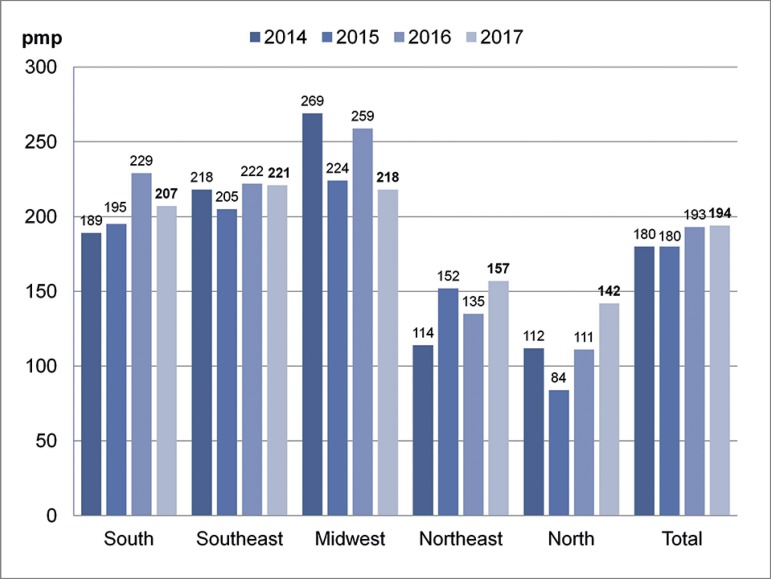
Estimated annual incidence of new dialysis patients per region in Brazil.

The number of patients starting treatment in 2017 in Brazil was estimated at 40,307, which yields an estimated incidence of 194 pmp (Figure 5) with rates ranging from 142 pmp in the North to 221 pmp in the Southeast. Estimated annual incidence was higher in Alagoas (340 pmp), Minas Gerais (282 pmp), and Distrito Federal (268 pmp), and lower in Maranhão (84 pmp), Pernambuco (84 pmp), and Amazonas (83 pmp). Almost half of the patients starting treatment in 2017 were in the Southeast region (48%). Incidence has been on the rise since 2012. The average annual estimated number of new patients is 28,392 from 2007 to 2012, and 37,024 from 2013 to 2017.

The incidence rate of new dialysis patients with diagnosis of diabetic nephropathy was 77 pmp (40% of incident patients), ranging from 49 pmp in the North to 112 pmp in the Midwest.

**Figure 6:**
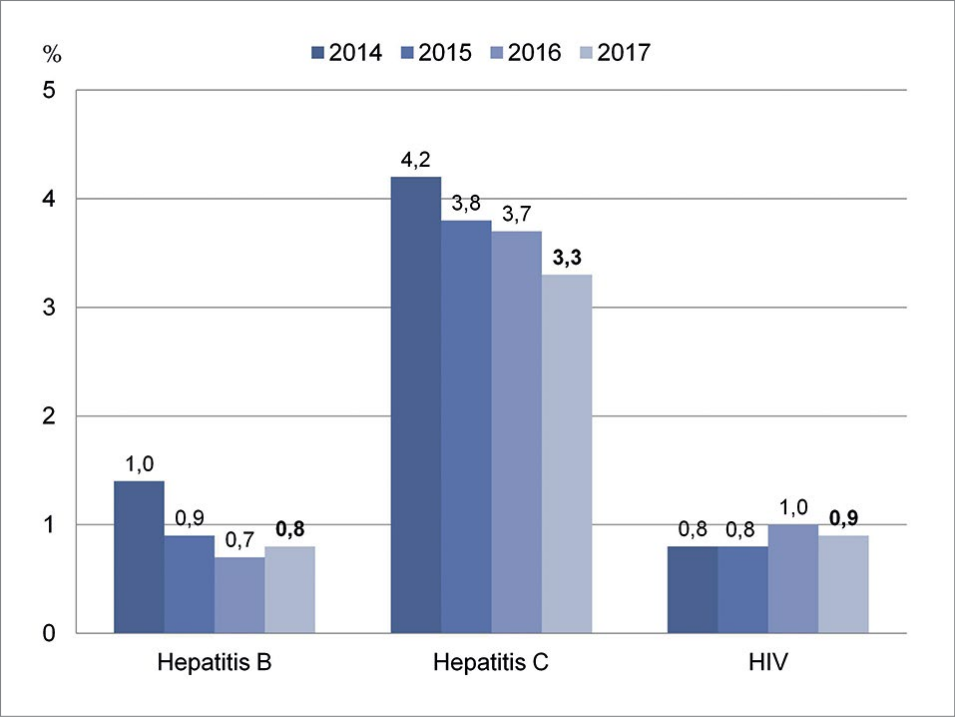
Prevalence of hepatitis B, hepatitis C, and HIV.

The prevalence of HIV-positive patients in chronic dialysis was 0.9% in 2017, a stable number in the last years (Figure 6). The proportion of positive serologic tests for hepatitis C and B among patients on chronic dialysis were respectively 3.3% and 0.8% in 2017. The prevalence of hepatitis C continued to decline.

The estimated proportion of patients on hemodialysis with a central venous catheter continued to increase, reaching 22.6% in 2017 (short-term catheters: 9.8%; long-term catheters: 12.8%). In 2017, 2.3% of the patients on hemodialysis used vascular grafts. The monthly hospitalization rate of the patients analyzed in July 2017 was 5.6%, similar to previous years.

**Figure 7:**
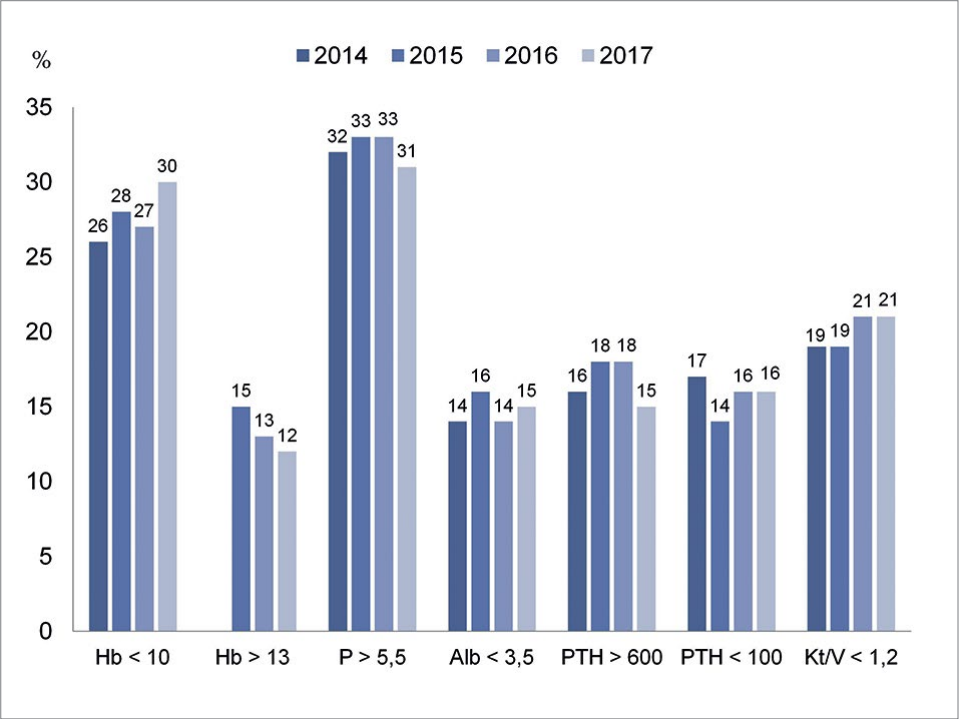
Proportion of patients on dialysis with laboratory results outside the levels recommended by KDIGO.

Figure 7 shows the proportions of patients on dialysis with laboratory results outside the levels recommended by KDIGO^2^. These proportions remain consistent along the recent years.

The proportion of patients using selected medications was also similar to recent years: 74% were on erythropoietin, 53% on intravenous iron, 22% on oral calcitriol, 8% on intravenous calcitriol, 2% on paricalcitol, 5% on cinacalcet, 40% on sevelamer, and 24% on calcium carbonate/acetate.

An estimated 31,226 patients were on the transplant waiting list in July 2017, the same proportion as in 2016 (24%).

**Figure 8:**
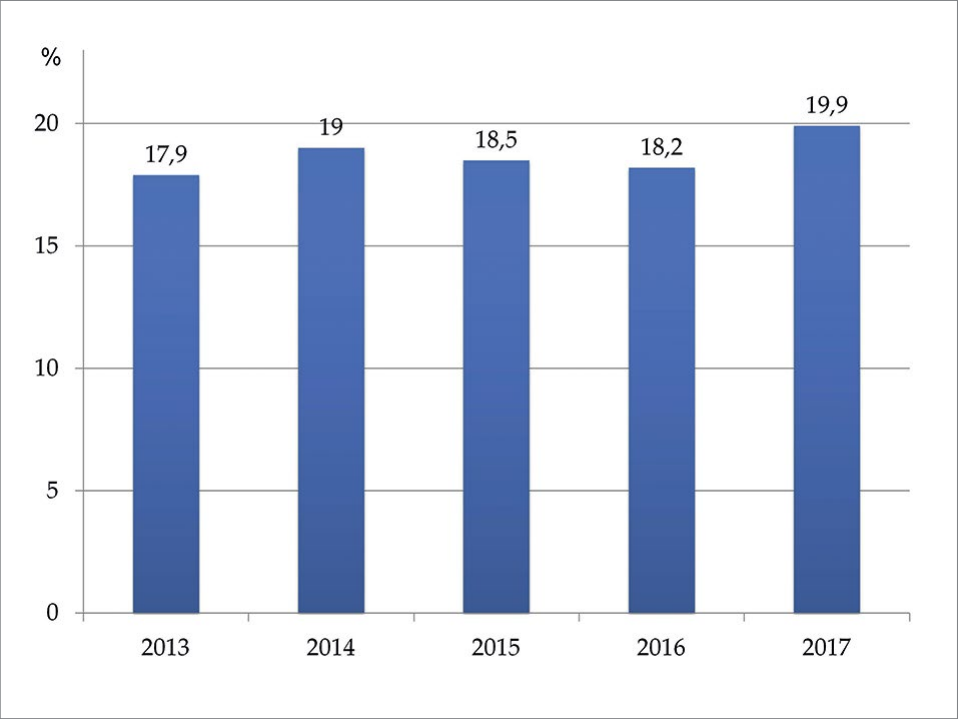
Annual estimated mortality rate of patients on dialysis.

The estimated number of deaths in 2017 was 25,187, yielding a gross death rate of 19.9% for the year (Figure 8).

## Discussion

The annual chronic dialysis survey has become a traditional event among Brazilian renal centers for the last 15 years,^3^^,^^4^ and this year 38% of centers joined the survey, representing a significant voluntary participation. The South and Southeast regions had response rates above the national average (40 and 44 %).

In the last 15 years, the number of end-stage renal disease patients on dialysis increased 4.2 times more than the number of active dialysis centers, increasing to 167 the average number of patients per center. Nonetheless, the average occupation rate is 85%. The average number of nephrologists per center was 6.

The growth rate of the number of patients on dialysis in Brazil is reducing. Although the annual increase of the estimated absolute number varies around 4,000 to 6,000 per year, the relative growth of dialysis population is decreasing as demonstrated when we compared percentage growth every five years.

The overall estimated prevalence increased 22.2% in 5 years, reaching 610 pmp. The actual prevalence of renal replacement therapy is the summation of the global prevalence of dialysis (610/pmp) and the prevalence of patients with functioning renal grafts (approximately 255/pmp in 2017), which yielded a rate around 865/pmp in 2017. This number was still lower than the prevalence reported in Chile (1,324/pmp), Uruguay (1,115/pmp), Western Europe (1000-1200/ pmp), the United States (2043/pmp) in 2016^5^, and Porto Rico (1,689 pmp), and similar to Argentina (895 pmp). The prevalence rate in Brazil was above the goal for Latin America set by SLAHN, which is 700 pmp by 2019. The rates concerning renal replacement therapy in the Southeast and South are probably close to 1000/pmp and, therefore, similar to values seen in developed nations. It is well known that there is a correlation between dialysis prevalence and gross domestic product (GDP). In the United States and other developed nations in Europe and Asia, the prevalence has increased steadily, although since the mid-2000s the incidence of patients on renal replacement therapy has flattened or grown marginally.^3^^,^^4^ For example, in the 2010-2015 period, standardized prevalence increased by less than 2% a year in the US.^5^

These annual estimates must be interpreted with caution due to the variation in the proportion of responding centers and the need to further validate the way in which the questions were answered. However, along the years of survey, the numbers seem to be consistent, and observed trends seem reliable. Rates have been higher in the Southeast, South, and Midwest and lower in the Northeast and North regions.

There is great variation between regions (and states) in Brazil. For the second year, we estimated the prevalence and incidence for each state, and the information was coherent for the most populous states, with higher prevalence rates in Minas Gerais, Rio de Janeiro, and Distrito Federal, higher absolute number of prevalent patients in São Paulo, Minas Gerais, and Rio de Janeiro, higher absolute number of incident (new) patients in São Paulo (8,889), Minas Gerais (5,957), and Paraná (2,765), and higher incidence rates in Alagoas, Minas Gerais, and Distrito Federal.

Similarly to prevalence, incidence varied significantly (142-221 pmp) between Brazilian regions. The actual rate of incident patients must include preemptive transplant recipients. The estimated global incidence of patients with chronic kidney disease on dialysis in Brazil was 194 pmp, similar to the numbers seen in many European countries, Uruguay, and Argentina, albeit lower than the rates observed in Puerto Rico (420 pmp), in the US (357/pmp), and Japan (286/pmp).^5^

The estimates indicated increases in incidence, especially in the North and Northeast, where the prevalence is lower, but is increasing fast. The average annual estimated number of new patients is increasing, being more than 40,000 for the first time. Two states (São Paulo and Minas Gerais) had almost 15,000 new patients (37%). For comparison, the number of new patients in the United States in 2017 was 123,688, more than 3 times the Brazilian figure. However, the American incidence has been stable for many years, while in Brazil it is rising.

Forty percent of the new patients starting dialysis had kidney disease reportedly due to diabetes, a proportion almost identical to the 2016 survey, greater than the numbers described for several European countries, and close to the levels found in the US (44%).^5^ This finding may indicate an increase in the contribution of diabetes among the causes of advanced chronic kidney disease, as indicated in previous reports.^3^^,^^4^ Nevertheless, diabetes accounts for 31% of prevalent dialysis patients. This paradox may be due to the higher mortality of these patients.

The proportion of children/adolescents and elderly patients (age ≥ 65 years) on dialysis in 2017 has not changed in relation to the percentages seen in 2016. The proportion of patients on maintenance hemodialysis and the number of patients covered by private health insurance on APD and daily hemodialysis was relatively unaltered in relation to previous years.^3^^,^^4^^,^^7^ The proportion of patients in the transplant waiting list remained 24%, close to figures from Argentina (28%) and Uruguay (20%). The use of peritoneal dialysis, home hemodialysis, and frequent hemodialysis remained substantially low.

The proportion of patients using venous catheters in hemodialysis grew considerably from 15.4% in 2013 to 22.6% in 2017.^6^ The data indicated that the growth was primarily related to increased use of long-term catheters (12.8%). The repercussions of this phenomenon need to be better studied.

Positive serologic tests for hepatitis B (0.8%) and HIV remained stable compared to previous years, while positivity for hepatitis C (3.3%) continued declining.

Concerning prescriptions, the proportion of patients taking selected medications was very similar to previous years, demonstrating consistency in the results. The most prescribed drugs were, in decreasing order, erythropoietin, intravenous iron, and sevelamer. Paricalcitol and cinacalcet were less used. Calcium-based phosphate binders have been used by 24% of the patients.

The gross death rate increased two points comparing to 2013 (17.9 to 19.9%)^3^^,^^4^. The proportions of patients with diabetic nephropathy and elderly individuals were unaltered since 2013, suggesting that overall mortality has not been affected by these factors.

The Brazilian economic crisis may explain the decreased proportion of new dialysis machines and increase of older ones, detected by the survey.

The voluntary nature of the survey, the grouping of patient data by dialysis center, and the lack of validation of the submitted answers require the inferences from this study to be drawn with caution.

## Conclusions

The Brazilian chronic dialysis survey has acquired relevance due to the consistency of its results year after year. The 2017 survey report showed continuing increase in the number of dialysis patients, although in a slower pace. Also of note was a slight increase in the estimated incidence rate. There is a significant inequality between states and regions in relation to these estimates, suggesting limitations to treatment access. The proportion of active dialysis centers increased less than the number of patients in the last 15 years. Diabetes was a major cause of entry in dialysis, although it did not represent the majority of patients undergoing treatment. Death rates increased slightly, and the use of venous catheters in hemodialysis has grown remarkably. Positive serologic test for hepatitis C continued declining. Our data may be used to establish policies for the care of the advanced CKD patient on dialysis in Brazil.
